# Deciphering the Binding Interactions between *Acinetobacter baumannii* ACP and β-ketoacyl ACP Synthase III to Improve Antibiotic Targeting Using NMR Spectroscopy

**DOI:** 10.3390/ijms22073317

**Published:** 2021-03-24

**Authors:** Sungjae Choi, Jungwoo Park, Jiwon Yeon, Ahjin Jang, Woo Cheol Lee, Yangmee Kim

**Affiliations:** Department of Bioscience and Biotechnology, Konkuk University, Seoul 05029, Korea; csj9897@konkuk.ac.kr (S.C.); jhopark123@konkuk.ac.kr (J.P.); jiwon4533@konkuk.ac.kr (J.Y.); ajin931017@konkuk.ac.kr (A.J.); wclee3@konkuk.ac.kr (W.C.L.)

**Keywords:** *A. baumannii*, fatty acid synthesis, acyl carrier protein, KAS III, protein–protein interaction (PPI) inhibitor, NMR spectroscopy

## Abstract

Fatty acid synthesis is essential for bacterial viability. Thus, fatty acid synthases (FASs) represent effective targets for antibiotics. Nevertheless, multidrug-resistant bacteria, including the human opportunistic bacteria, *Acinetobacter baumannii,* are emerging threats. Meanwhile, the FAS pathway of *A. baumannii* is relatively unexplored. Considering that acyl carrier protein (ACP) has an important role in the delivery of fatty acyl intermediates to other FAS enzymes, we elucidated the solution structure of *A. baumannii* ACP (AbACP) and, using NMR spectroscopy, investigated its interactions with β-ketoacyl ACP synthase III (AbKAS III), which initiates fatty acid elongation. The results show that AbACP comprises four helices, while Ca^2+^ reduces the electrostatic repulsion between acid residues, and the unconserved F47 plays a key role in thermal stability. Moreover, AbACP exhibits flexibility near the hydrophobic cavity entrance from D59 to T65, as well as in the α_1_α_2_ loop region. Further, F29 and A69 participate in slow exchanges, which may be related to shuttling of the growing acyl chain. Additionally, electrostatic interactions occur between the α_2_ and α_3_-helix of ACP and AbKAS III, while the hydrophobic interactions through the ACP α_2_-helix are seemingly important. Our study provides insights for development of potent antibiotics capable of inhibiting *A. baumannii* FAS protein–protein interactions.

## 1. Introduction

The World Health Organization (WHO) reported the six most notorious drug-resistant pathogens responsible for causing infections in hospital environments and the community. The list included *Enterococcus faecium*, *Staphylococcus aureus*, *Klebsiella pneumoniae*, *Acinetobacter baumannii*, *Pseudomonas aeruginosa*, and *Enterobacter* species (ESKAPE) [1,2]. *A. baumannii* is a nosocomial ESKAPE pathogen with a high multidrug resistance (MDR) incidence rate [3,4]. Gram-negative bacterium, including *A. baumannii*, contain a lipopolysaccharide (LPS) layer comprising the primary hydrophobic outer lipid membrane, which serves to protect the bacterial cell against chemical attack while imposing toxicity to the host. Furthermore, *A. baumannii* contains many types of outer membrane proteins, including porins, OmpA, carboxylate channels, and efflux pumps, which function in virulence and permeability, making the development of novel antibiotics against *A. baumannii* challenging. Due to the rapid emergence of antibiotic resistance in *A. baumannii*, researchers have begun to focus on developing alternative strategies for treating MDR *A. baumannii* [5,6,7].

Gram-negative bacteria are generally more difficult to treat with common antibiotics than Gram-positive bacteria, due to the differences in cell wall composition. *A. baumannii*, specifically, has developed considerable resistance to antibacterial agents, including cephalosporins, penicillin, fluroquinolones, and aminoglycoside agents [8,9]. Moreover, with the recent increase in carbapenem use against Gram-negative infection, an emergence of MDR *A. baumannii* has been reported, indicating a resistance to most available antibacterial agents [10]. Due to the resulting increase in the number of infections caused by MDR *Acinetobacter* strains, the research focus has been placed on developing new effective antibiotics against *A. baumannii.*

Considering the ever-increasing prevalence of MDR *A. baumannii* infections, strategies for developing novel antibacterial targets are needed. One such strategy is targeting proteins in pathways that are essential for bacterial viability, such as those involved in fatty acid synthesis. Fatty acids are critical to the survival of all organisms, as they are used for storing energy and forming cell membranes while also functioning as intermediates in various signaling pathways [11]. Moreover, the fatty acid synthesis system employs fatty acid synthases (FASs) to produce fatty acids of various lengths that serve as building blocks for the cell membrane. Typically, these systems are divided into two groups, type I and type II [12]. Bacterial type II FAS systems are composed of small proteins, each of which generally catalyzes individual steps in the FAS reaction [13]. One such important protein is β-ketoacyl acyl carrier protein (ACP) synthase (KAS III), which produces acetoacyl ACP from malonyl-ACP using acetyl-Coenzyme A(CoA) as a substrate in the initiation step of the FAS elongation cycle. Hence, KAS III represents an effective target for novel antibiotics to inhibit the FAS system. Accordingly, we previously designed antimicrobial inhibitors for the KAS III proteins of various pathogens [14,15]. In particular, we determined the tertiary structures of FAS proteins from *A. baumannii* and sought to identify inhibitors for the protein β-ketoacyl ACP synthase (AbKAS III) [16,17,18,19]. Since nuclear magnetic resonance (NMR) spectroscopy is very useful in the early stage of target-based drug discovery, we utilized NMR to investigate the molecular interactions between the FAS proteins in the current study.

Acyl carrier proteins (ACPs), which are highly conserved among various organisms, are small acidic proteins (9 kDa) with a pI value of approximately 4.0 [12]. ACPs play an important role in both type I and II FAS systems by binding the growing acyl chain in its hydrophobic cavity and, subsequently, delivering fatty acid intermediates, of various lengths, to other FAS enzymes [20]. Moreover, ACPs also play a key role in the polyketide synthesis system and nonribosomal peptide synthesis system (NRPSP), which are catalyzed by polyketide synthases (PKSs) and nonribosomal peptide synthetases (NRPs), respectively [21,22]. To carry out their functions by interacting with PKSs and NRPSs, ACPs must be structurally flexible and mobile proteins [23]. All ACPs are composed of four-helix bundles connecting three loop regions, as well as a conserved serine residue at the front of α_2_-helix that attaches to a 4′-phosphopantetheine prosthetic group (4-PP) in the DSL (Asp–Ser–Leu) motif [12,24]. In particular, the α_2_-helix of ACPs, referred to as the “recognition α_2_-helix”, contains acidic residues and is important for interactions with FAS enzymes [13,25]. NMR spectroscopy has been successfully applied for the identification of the resolution structures of type II ACPs in *Escherichia coli* (EcACP) [26,27,28], *Vibrio harveyi* (VhACP) [29], *Borrelia burgdorferi* (BbACP) [30], *Enterococcus faecalis* (EfACP) [31], and *Thermotoga maritima* (TmACP) [32].

To effectively target FAS for the development of antibiotics, a clear understanding of the structures of ACPs in solution, as well as of their binding dynamics to the bacterial FAS proteins, is required. While this has been elucidated for the ACPs of many bacterial species, that of *A. baumannii* (AbACP) remains unknown. Hence, the current study sought to not only elucidate the solution structure of AbACP by NMR spectroscopy but also investigate its thermal stability and interactions with AbKAS III, a protein responsible for initiating fatty acid elongation. This study will be helpful to gain insights for the development of new FAS targeting protein–protein interaction (PPI) inhibitors for *A. baumannii*.

## 2. Results

### 2.1. Comparison of Pathogenic ACP Sequences

**Figure 1:**
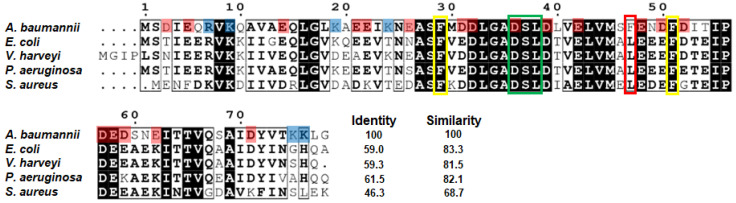
Comparison of bacterial acyl carrier protein (ACP) sequences. The sequence alignment of the Gram-negative bacteria (*Acinetobacter baumannii, Escherichia coli, Vibrio harveyi*, and *Pseudomonas aeruginosa*) and Gram-positive bacteria (*Staphylococcus aureus*). The 100% conserved residues are shown in black boxes, and residues with a global score of similarity from 0 to 1.0 above 0.7 are shown in black boxes without shading. Based on *A. baumannii* ACP (AbACP), the identity and similarity of each ACP is marked. The conserved Asp–Ser–Leu (DSL) motif is marked in a green box, and the conserved phenylalanines are marked in a yellow box, while the unconserved F47 in AbACP is marked in a red box. Negatively charged residues and positively charged residues in AbACP are marked with red and blue shading, respectively.

Multiple sequence alignment of AbACP with *E. coli* ACP (EcACP) and other pathogenic bacterial ACPs showed that the overall AbACP sequence shows high sequence similarity with most other bacterial ACPs (Figure 1), implying that the sequence of AbACP is highly conserved among pathogenic bacteria. In particular, AbACP shares high identity and similarity with Gram-negative bacterial ACPs (*E. coli*: 59.0% and 83.3%, *V. harveyi*: 59.3% and 81.5%, and *P. aeruginosa*: 61.5% and 82.1%, respectively) compared to Gram-positive ACPs (*S. aureus*: 46.3% and 68.7%). All pathogenic bacterial ACPs, including AbACP, contain a serine residue as the 4-PP group attachment site for shuttling growing acyl chains. Moreover, 4-PP attachment induces large chemical shift perturbations (CSP) near S37 and the hydrophobic cavity (Figure S1). The conversion of apo-AbACP to the holo form upon 4-PP prosthetic group attachment resulted in large CSPs (0.1 ppm ≤ CSP < 0.4 ppm) for the residues near the prosthetic group attachment site, as well as the residues forming the hydrophobic cavity (S37, L38, V41, M45, and A69) (Figure S1). As shown in Figure 1, the “recognition α_2_-helix” is generally highly conserved among bacterial ACPs. Moreover, ACP is highly acidic in α_2_-helix and α_3_-helix, with strong electrostatic repulsion between the negatively charged residues. Meanwhile, AbACP has an unconserved additional phenylalanine (F47 marked in the red box) in the middle of α_2_-helix corresponding to Leu in other mesophilic bacterial pathogenic ACP (Figure 1). This Leu in EcACP is reportedly important to open up a path into the binding cavity of ACP [20]. Interestingly, hyperthermophilic *Thermotoga maritima* ACP has phenylalanine at this position [32]. In this study, we investigated the solution structure of AbACP and the role of these residues in the structure and thermal stability of AbACP in comparison with other ACPs.

### 2.2. Solution Structure of AbACP

**Figure 2:**
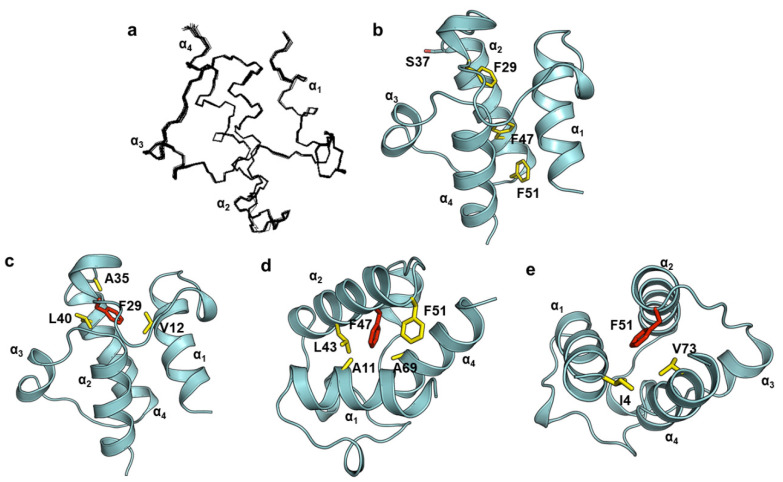
The structure of AbACP (PDB ID: 7E42). (**a**) The superimposed backbone atoms of the 20 lowest energy structures of AbACP. (**b**) Hydrophobic interactions built by three phenylalanine molecules in the hydrophobic cavity. Hydrophobic contacts formed by the conserved F29 (**c**), unconserved F47 (**d**), and conserved F51 (**e**).

We determined the solution structure of holo-AbACP using NMR spectroscopy, and the summary of the structural statistics is presented in Table 1. A total of 1465 NMR restraints were detected, comprising 1176 nuclear Overhauser effect (NOE) distance restraints, 141 backbone torsion-angle restraints, 74 orientational restraints, and 74 hydrogen bond restraints, all of which were used to calculate the solution structure of AbACP. The RMSD (root mean square deviation) values of AbACP for the backbone and heavy atoms in all residues were 0.1 Å and 0.4 Å, respectively. The superimposed 20 lowest energy tertiary structures are shown in Figure 2a. The AbACP structure is composed of four helices similar to other bacterial ACPs: α_1_: E5-L16, α_2_: S37-F51, α_3_: D57-S60, and α_4_: V66-K76, connected by three loop regions. Additionally, three phenylalanines are present in the AbACP sequence and represent the key residues in the hydrophobic cavity, which accommodates the growing acyl chains (Figure 2b). Specifically, the conserved F29 positioned at the flexible α_1_α_2_ loop forms a hydrophobic interaction with V12, A35, and L40, thereby stabilizing the long α_1_α_2_ loop (Figure 2c). Meanwhile, the unconserved F47 (Figure 2d) mediates the formation of additional hydrophobic interactions with A11, L43, F51, and A69. As commonly observed in other bacterial ACPs, AbACP contains a hydrophobic triad formed by I4, F51, and V73 at the top of the hydrophobic cavity, which ensures that the helices of the hydrophobic cavity remain in close proximity to each other (Figure 2e).

### 2.3. Thermal Stability of AbACP and the Effect of Metal on Its Stability

**Figure 3:**
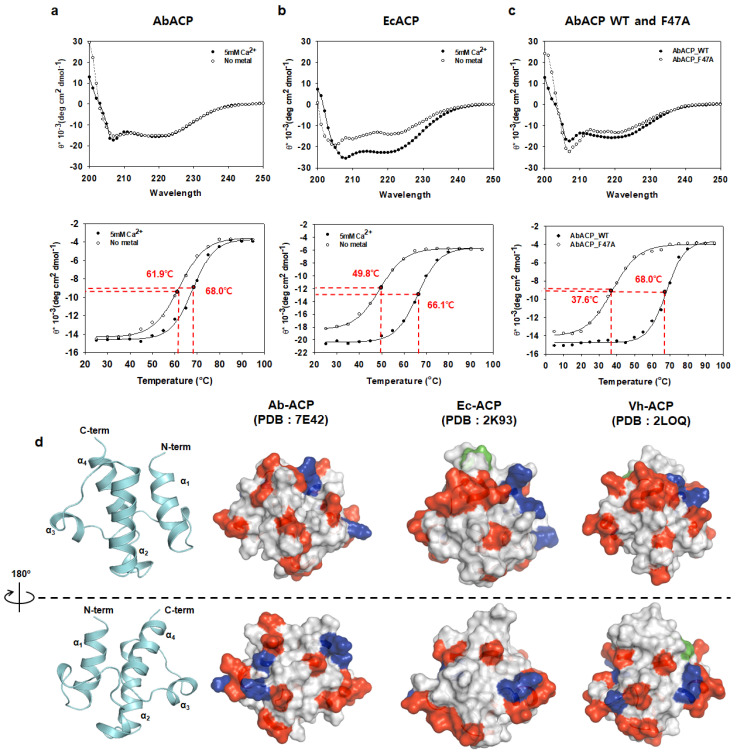
The circular dichroism (CD) spectra and distribution of surface charges in AbACP compared to those of Gram-negative bacterial ACPs. (**a**) CD spectrum of AbACP from 200 to 250 nm in wavelength and its melting curves from 25 °C to 95 °C in the presence and absence of Ca^2+^. (**b**) CD spectrum of *Escherichia coli* ACP (EcACP) and its melting curves from 25 °C to 95 °C in the presence and absence of Ca^2+^. (**c**) CD spectra of wild-type (WT) AbACP and F47A mutants and their melting curves in the presence of Ca^2+^. (**d**) Surface charge distribution of AbACP in comparison with those of EcACP and *Vibrio harveyi* ACP (VhACP). Negatively charged residues (Glu and Asp) are indicated in red, and positively charged residues (Arg and Lys) are shown in blue. His are colored in green.

As shown in the sequence alignment, there are many negatively charged and fewer positively charged residues on bacterial ACPs, resulting in electrostatic repulsion at the surface [29]. The “recognition α_2_-helix”, which consists of conserved negatively charged residues, is important for the interaction with other FAS enzymes [13,36]. Considering that AbACP has 19 negatively charged residues and only six positively charged residues, it exhibits a higher stability when divalent cations are present to release the electrostatic repulsion. Hence, to investigate the effect of metal ions on the thermal stability of AbACP, we conducted circular dichroism (CD) experiments. The CD spectra of AbACP from 200 to 250 nm showed double minima at 205 nm and 222 nm, which is characteristic of α-helical structures and agrees with the solution NMR structure (Figure 3a). Moreover, the thermal denaturation curves of AbACP from 25 °C to 95 °C (Figure 3a) showed that the melting temperature (T_m_) of AbACP is 68.0 °C in the presence of Ca^2+^, which is similar to that of EcACP (66.1 °C; Figure 3b). In contrast, in the absence of Ca^2+^, AbACP has a melting temperature (61.9 °C) that was 12 degree higher than that of EcACP (49.8 °C). Therefore, AbACP may have higher structural stability compared EcACP in the absence of divalent cations. Additionally, in the presence of Ca^2+^, the electrostatic repulsion in both proteins was reduced; however, in the absence of metal ions, the repulsion between the negatively charged residues in EcACP caused a more significant destabilization of the protein structure compared to AbACP. This is likely due to the additional positively charged surface residues on AbACP resulting in reduced repulsion in comparison with EcACP and VhACP (Figure 3d).

To investigate the importance of the unconserved F47, we measured the thermal stability of the F47A mutant compared to that of WT AbACP. The CD data showed that the T_m_ of F47A decreased dramatically to 37.6 °C, accounting for a 30-degree difference compared to WT AbACP (Figure 3c), implying that hydrophobic interaction mediated by F47 is important for the thermal stability of the AbACP structure.

### 2.4. Backbone Dynamics of AbACP

**Figure 4:**
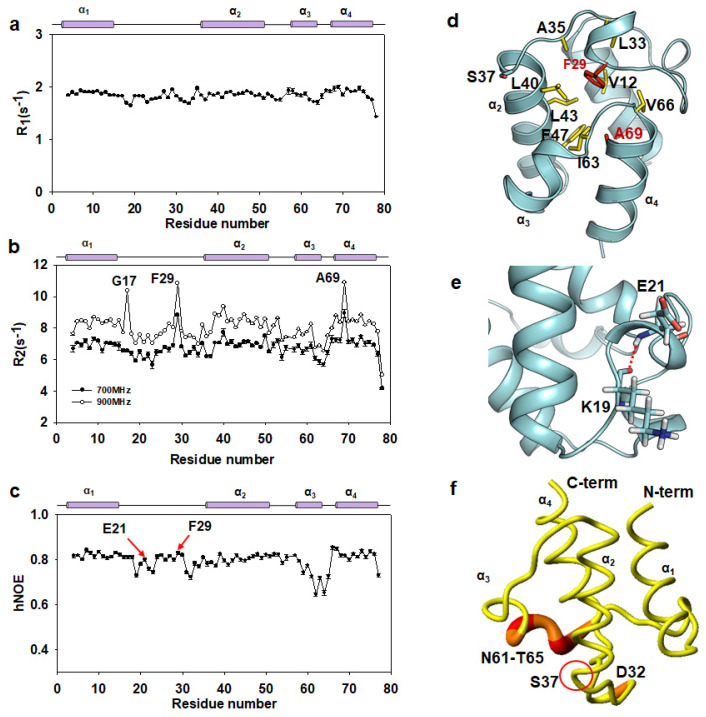
Backbone dynamics of AbACP. (**a**) The longitudinal (R_1_), (**b**) transverse (R_2_), and (**c**) heteronuclear nuclear Overhauser effect (hNOE) of AbACP. R_1_ and hNOE relaxation experiments were performed at 25 °C using a Bruker Advance 700-MHz spectrometer, while R_2_ experiments were performed at two different field strengths, 700 MHz and 900 MHz. Above the plot, the filled purple bars represent the α-helices of AbACP. (**d**) F29 and A69 with slow motions in the R_2_ relaxation rates are shown in the red stick model, and the residues forming hydrophobic contacts with F29 and A69 in the hydrophobic cavity are shown in the yellow stick model. (**e**) Hydrogen bonding between K19 and E21 contributes to the rigidity of E21 in the middle of the α_1_α_2_ loop. (**f**) Residues showing flexibility with low hNOE value rendering in the structure of AbACP. Residues with hNOE < 0.73 are shown with larger ribbon diameters colored from orange to red. S37, which is the 4′-phosphopantetheine prosthetic (4-PP) attachment site, is marked in Figure 4d,f.

To investigate the motional properties of AbACP, we conducted backbone dynamic experiments for AbACP (Figure 4a−c). Longitudinal (R_1_), transverse (R_2_), and heteronuclear NOE (hNOE) experiments on a picosecond-to-nanosecond time scale were analyzed, as described previously [31,37]. The overall averages of the R_1_, R_2_, and hNOE values for AbACP were as follows: 1.84 ± 0.02 s^−1^, 6.78 ± 0.12 s^−1^, and 0.79 ± 0.01 s^−1^, respectively. The average R_2_/R_1_ ratio for AbACP was 3.69, implying the existence of a monomer structure in the solution. Moreover, the R_1_ rates demonstrated that the rapid time scale motions, on a nanosecond–picosecond time scale, were uniform throughout the sequence.

Spin–spin(R_2_) relaxation rates can detect slow motions in the millisecond-to-microsecond time scale. Certain ACPs have been reported to show conformational exchanges, particularly in the loop region related to acyl chain shuttling [30,31,38], whereas most ACPs, such as EcACP, show only picosecond-to-nanosecond fast time scale motions [37]. As shown by the R_2_ relaxation rates at the 700-MHz magnetic field (Figure 4b), F29 and A69 exhibit slow motions in AbACP. A conformational exchange may contribute to the transverse relaxation rate (R_2_) and can show the dependence on the static magnetic field strength [39,40]. Therefore, we measured the R_2_ relaxation rates at two different field strengths, 700 MHz and 900 MHz, to confirm the possible chemical exchange in µs–ms time scale motions for F29 and A69 (Figure 4b). The results showed that the overall R_2_ rates for all residues in Figure 4b increased in a field-dependent manner. Especially, F29 and A69 showed even higher R_2_ rates at 900 MHz compared to those at 700 MHz, implying that they may be in a conformational exchange in the slow time scale due to participation in hydrophobic contact with residues or the 4-PP prosthetic group in the hydrophobic cavity to help sequester the growing acyl chains, as well as the prosthetic group attached to S37 (Figure 4d). Interestingly, G17 at the beginning of the flexible α_1_α_2_ loop showed a high R_2_ rate only at 900 MHz which needs to be investigated further in our future study. 

Additionally, the long α_1_α_2_ loop showed low hNOE values, reflecting the loop flexibility, while the hydrogen bonding between K19 and E21 contributes to the rigidity of E21 in the center of the α_1_α_2_ loop (Figure 4e). The F29 in the center of the α_1_α_2_ loop also showed high hNOE, reflecting stable hydrophobic interactions in the cavity (Figure 4c,d). Furthermore, the α_3_-helix and α_3_α_4_ loop regions (D59-T65), which form the entrance of the hydrophobic cavity, had significantly lower hNOE values compared to the averages for the α_1_, α_2_, and α_4_-helices. Spin–relaxation data further indicated that the high flexibility of the α_1_α_2_ and α_3_α_4_ loops facilitate the shuttling of growing acyl chains into the hydrophobic pocket (Figure 4c,f).

### 2.5. Mapping the Binding Residues of AbACP to AbKAS III by Chemical Shift Perturbation

Type II FAS utilizes individual proteins participating stepwise in fatty acid synthesis. Meanwhile, ACP interacts with various FAS enzymes to deliver growing acyl chains during fatty acid synthesis [41,42]. Considering that the inhibition of PPIs between ACP and FAS enzymes represents an attractive approach for the development of novel antibiotics, an in-depth analysis of the binding interactions between ACP and FAS enzymes must be performed. Specifically, since KAS III is a key enzyme that initiates the fatty acid elongation process by condensing 2-carbon units from acyl-CoA and malonyl-ACP, the binding interaction between malonyl-AbACP and AbKAS III was investigated by NMR spectroscopy to provide insights for the development of novel PPI inhibitors as effective antibiotics against *A. baumannii*.

**Figure 5:**
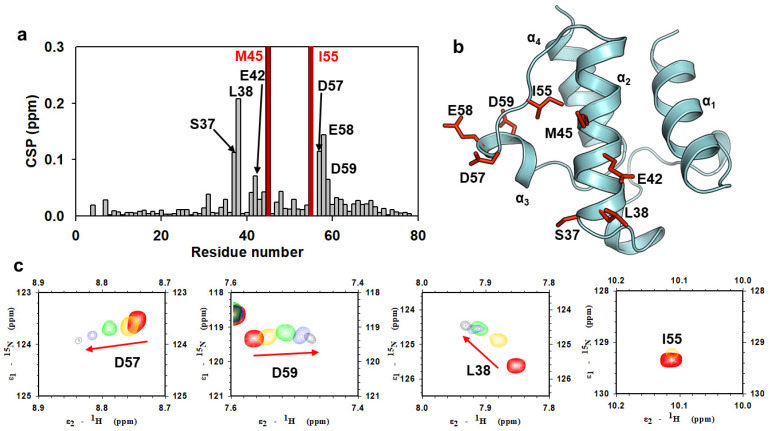
The chemical shift perturbation of malonyl-AbACP binding to β-ketoacyl ACP synthase III (AbKAS III). (**a**) The chemical shift perturbation of malonyl-AbACP in ^1^H-^15^N HSQC-TROSY spectra upon titration with AbKAS III from a 1:0 to 1:1.5 ratio. Chemical shift perturbation (Δδ) is calculated using the equation Δδ = (0.5(Δδ(^1^H)^2^ + (αΔδ(^15^N)^2^)))^1/2^ (α = 0.2 for most residues, and α = 0.14 for glycine) [43]. (**b**) Structural representations of residues showing large chemical shift perturbations (CSP) (CSP > 0.05) or peak disappearance upon AbACP titration with AbKAS III. (**c**) Peak traces or disappearance of the residues in the ^1^H-^15^N HSQC-TROSY spectra of AbACP upon titration with AbKAS III at the ratios of 1:0 (black), 1:0.1 (yellow), 1:0.5 (cyan), 1:1 (green), and 1:1.5 (red).

To determine the binding residues of AbACP to AbKAS III, we performed an NMR experiment and monitored the CSPs, or changes in peak intensities, for ^1^H-^15^N Heteronuclear Single Quantum Coherence(HSQC)–Transverse Relaxation-optimized Spectroscopy (TROSY) spectra upon the titration of AbKAS III to malonyl-AbACP. The amide signals of the AbACP residues that interact with AbKAS III gradually shifted in one direction or disappeared. The CSP of these residues was calculated using the equation reported by Wuthrich et al. and represented in Figure 5a [43]. The residues affected by KAS III binding (CSP > 0.05 ppm or peak disappearance) were mapped on the structure of AbACP in Figure 5b. The peak traces for these malonyl-AbACP residues in the ^1^H-^15^N HSQC-TROSY spectra showed that peak movement was observed due to AbKAS III binding (Figure 5c). These results imply that exposed negatively charged residues (E42, D57, E58, and D59) in α_2_- and α_3_-helices of AbACP exhibited primarily large CSP by forming electrostatic interactions with AbKAS III. Since the malonyl group is attached to the S37, a large CSP was also observed near S37. Furthermore, L38 showed a large CSP and amide peaks for M45, which disappeared during titration, implying that they may form hydrophobic interactions with AbKAS III. The amide peaks of M45 at α_2_-helix and I55 at the flexible α_2_α_3_-loop region disappeared after the second titration with a ratio of 1:0.1 due to line broadening caused by the chemical exchanges between the free and bound forms (Figure 5c).

### 2.6. Binding Model of AbACP to AbKAS III

**Figure 6:**
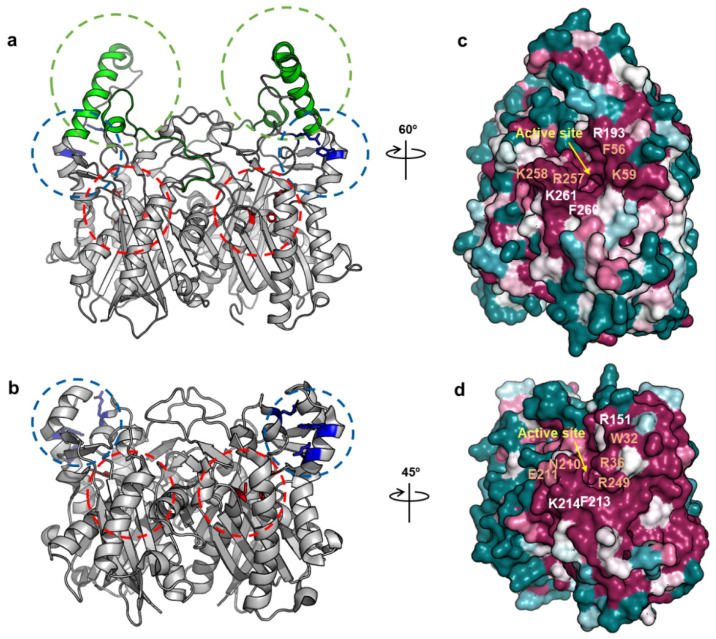
Overall structure and conserved residues of AbKAS III and EcKAS III. (**a**) Homodimer X-ray structure of AbKAS III (5YO9) [17]. Two inserted sequences in AbKAS III are marked in green, the CoA-binding site is marked in blue, and the catalytic triad residues at the active site are marked in red. (**b**) X-ray structure of EcKAS III (1EBL) [44]. Colors in circles have the same notion as described for Figure 6a. (**c**) The conserved residues of AbKAS III in the ConSurf model are calculated based on the PROTEIN BLAST analysis for the proteins with high sequence similarity to AbKAS III (dark green, variable, white, normal, and dark purple, conserved) [45]. Entrance of 4-PP or the malonyl group is indicated by a yellow arrow. Important residues that are conserved in all KAS III in Figure 7 are labeled in white, while residues that are not conserved in AbKAS III and EcKAS III are labeled in orange. (**d**) Conserved residues of EcKAS III in the ConSurf model calculated based on the PROTEIN BLAST analysis for the proteins with high sequence similarity to EcKAS III without two insertions (dark green, variable, white, normal, and dark purple, conserved) [45]. Entrance of 4-PP or the malonyl group is indicated by a yellow arrow. Important residues conserved in all KAS III are labeled in white, and residues that are not conserved in AbKAS III and EcKAS III are labeled in orange.

**Figure 7:**
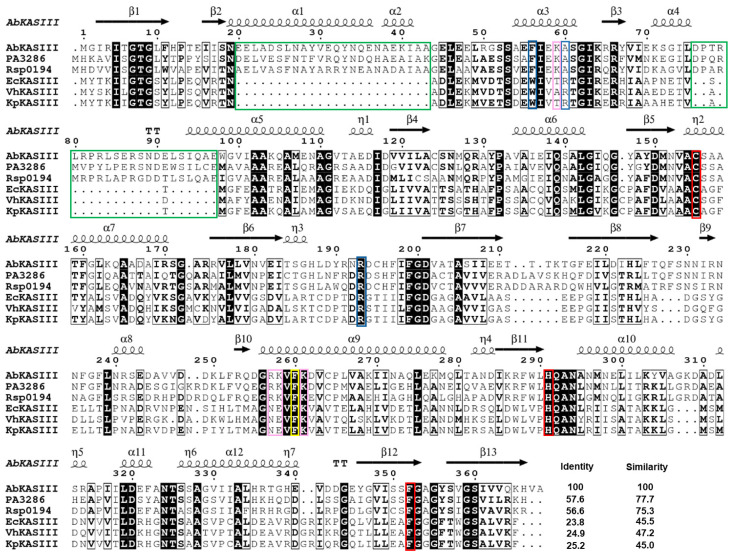
Multiple sequence alignment of AbKAS III. KAS III from *A. baumannii*, PA3286 (KAS III ortholog of *P. aeruginosa*), Rsp0194 (*Ralstonia solanacearum*), *E. coli*, *V. harveyi*, *and K. pneumoniae* are aligned. Two inserted sequences in AbKAS III are highlighted with a green box. The CoA-binding sites are marked in blue, and the catalytic triad residues at the active site are marked in red. Conserved hydrophobic phenylalanine (F260 in AbKAS III), which is involved in hydrophobic interactions with ACP, are highlighted in a yellow box. Positively charged residues near the active site, conserved only in KAS III with two insertions, are highlighted in pink.

To understand the molecular interactions between AbACP and AbKAS III, a docking simulation was performed to construct a binding model of the ACP-KAS III complex based on our NMR data mapping the binding residues in AbACP. We reported previously the crystal structure of AbKAS III, and it forms a homodimer displaying the typical KAS III structure [17]. However, AbKAS III shows two extra distinct insertion sequences marked in green in Figure 6a. compared to the X-ray structure of EcKAS III (Figure 6b) [17,44]. These extra insertions contain the α_1_, α_2_, and α_4_-helices of AbKAS III. Similar to other KAS III, AbKAS III has a catalytic triad (Cys–His–Asn triad) in the active site marked in red (Figure 6a). The CoA-binding site of AbKAS III has two residues (F56 and R193) marked in blue (Figure 6a). Additionally, Figure 6c shows the ConSurf surface model [45] calculated based on the PROTEIN BLAST analysis for proteins with a high sequence similarity to AbKAS III. Residues near the active sites are highly conserved. The sequence alignment of the bacterial KAS III proteins (Figure 7) in comparison to that of AbKAS III revealed that AbKAS III shares a high sequence homology with the KAS III of pathogenic bacteria, such as PA3286 (KAS III ortholog of *P. aeruginosa*) [46] and *Ralstonia solanacearum* KAS III. They have two extra insertion sequences with over 70% sequence similarity, which is absent in the KAS III from *E. coli, Vibrio harveyi, and K. pneumoniae*. These two extra insertions contain the α_1_, α_2_, and α_4_-helices (Figure 7). Moreover, the key residues in the CoA-binding site of AbKAS III differ from those of EcKAS III; AbKAS III has two residues (F56 and R193) in the CoA-binding site, whereas EcKAS III has three residues (W32, R36, and R151) (Figure 6a,c and Figure 7). R193 and F56 in AbKAS III corresponding with W32 in EcKAS III are the key residues in the CoA-binding site by forming π-stacking interactions with the purine ring of CoA. In the CoA-binding site, AbKAS III contains F56, similar to the homolog proteins with two insertion sequences, while the KAS III without inserts contain tryptophan. In contrast, R193 in the CoA-binding site of AbKAS III is conserved as R151 in EcKAS III. In our previous study, the mutation of F56 in AbKAS III caused a reduced acceptance of longer acyl-CoA, such as octanoyl-CoA [17]. A conformation–sensitive assay confirmed that the reactivity of the F56W mutant was decreased by 40% compared to the wild type [17]. C112, H244, and N274, which formed the catalytic triad in EcKAS III corresponding to C155, H291, and N324 of AbKAS III, were highly conserved (Figure 6a,c and Figure 7).

**Figure 8:**
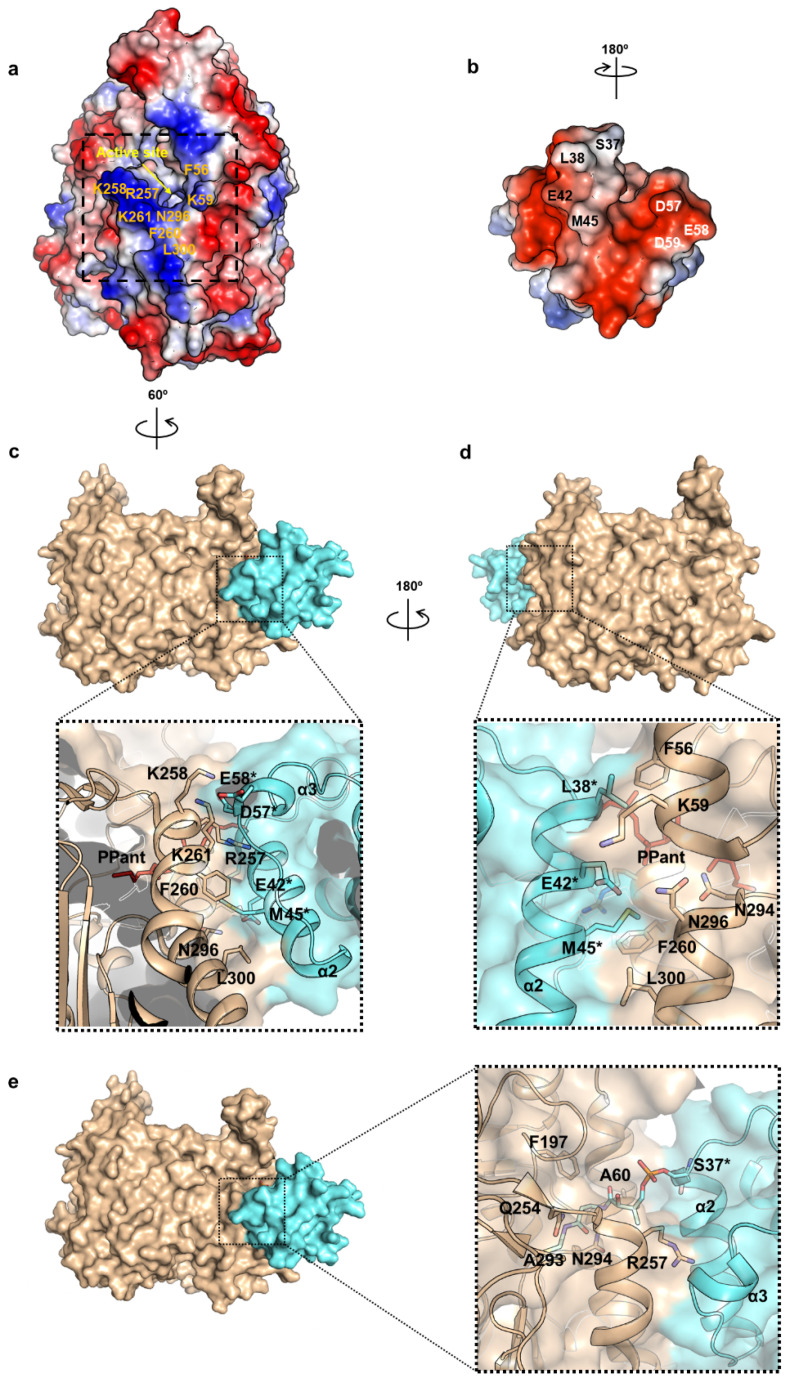
Electrostatic potential surface plot and binding model for AbKAS III and AbACP. (**a**) Electrostatic potential surface plot of AbKAS III. Residues near the ACP-binding site of AbKAS III in the black box are labeled in orange. (**b**) Electrostatic potential surface plot of AbACP depicted at twice the enlarged scale compared to AbKAS III. Residues in the α_2_-helix with large CSPs due to interactions are labeled in black, and residues in the α_3_-helix with large CSPs are labeled in white. With 180-degree rotation, the residues labeled in ACP bind to the residues in AbKAS III marked with black lines in Figure 8a. (**c**) Surface rendering of the AbKAS III–AbACP complex docking structure. Detailed view of the interactions between AbKAS III and AbACP with translucent surface rendering is displayed in the inset figure. Residues of ACP are also labeled with asterisks. (**d**) Surface rendering of the docking structure and the interactions between AbKAS III and AbACP with translucent surface rendering with 180-degree rotation of Figure 8c. AbKAS III is colored in beige, and ACP is colored in cyan. Structure of the 4-PP prosthetic group attached to the S37 of AbACP in the active site cavity of AbKAS III is depicted with a red stick model. Residues involved in the interaction are labeled in black, and the residues of ACP are also labeled with asterisks. (**e**) Surface rendering of the docking structure and the interactions between AbKAS III and the 4-PP prosthetic group attached to S37 in ACP.

Important residues near the active site of AbKAS III, as well as residues in the α_2_ and α_3_-helices in ACP with large CSP upon binding to AbKAS III, are marked in Figure 8a,b. Meanwhile, the positively charged electrostatic surface of AbKAS III can bind to the negatively charged surface of AbACP (Figure 8b) with a 180-degree rotation. F260 and K261 are highly conserved residues in all KAS III (Figure 7), while F56, K59, R257, and K258 are conserved only in those KAS III with two insertions. Similar to the previously reported EcACP-KAS III docking model, F260 in AbKAS III and M45 in AbACP, which are highly conserved in all bacterial FASs, form important hydrophobic interactions [36]. As shown in Figure 7, A60 and N296 of AbKAS III, which correspond to R36 and R249 near the entrance of EcKAS III, are not conserved, while R257 and K258 located at the entrance of the AbKAS III active site, corresponding to N210 and E211 in EcKAS III, are conserved only in KAS III with two insertions. Due to the differences in the KAS III residues that interact with ACP during the elongation process, it is necessary to understand the differences in their interactions with ACP and KAS III from different species to facilitate the development of antibiotics with high specificity. 

Based on the binding energy, as well as the agreement with CSPs measured from NMR titration experiments, we selected the best binding model among 100 docking runs. The docking model with the lowest binding energy (8.368 kcal/mol), where the 4-PP prosthetic group attached to S37 penetrates into the active site cavity of AbKAS III, showed the highest population (41%) among all clusters and showed good agreement with the experimental CSP data (Figure 8). The docking model indicates that the α_2_-helix, as well as the α_3_-helix in AbACP, form important binding interactions with AbKAS III, as listed in Table S1 (Figure 8c−e). Specifically, L38 and M45 in the α_2_-helix of ACP form hydrophobic interactions with F56 and F260 in AbKAS III, respectively (Figure 8c,d). Furthermore, the 4-PP prosthetic group attached to S37 in ACP has interactions with A60, F197, Q254, R257, A293, and N294 in AbKAS III (Figure 8e). The hydrophobic interactions between M45 in AbACP and F260 in AbKAS III, corresponding to the interactions between M45 in EcACP and F213 in EcKAS III in the previously reported EcACP-EcKAS III docking model, might be highly conserved in all bacterial FAS [36]. Although R261 and K303 in AbKAS III are well-conserved ACP-binding residues among all KAS III proteins, certain unconserved interactions, such as electrostatic interactions between unconserved N296 in AbKAS III and E42 in AbACP, have been newly observed. Positively charged K59, R257, and K258 in AbKAS III, which appeared only in KAS III with two insertions, are the key residues that form electrostatic interactions with E42 in the α_2_-helix of ACP, as well as with D57 and E58 in the α_3_-helix of AbACP, respectively (Figure 8c). Additionally, K261 in AbKAS III forms electrostatic interactions with E58 in the α_3_-helix of AbACP. Hence, the α_2_ and α_3_-helices in AbACP serve as the major interacting regions of AbACP-AbKAS III, whereas it was previously reported that the α_1_-helix and α_2_-helix of EcACP interact with EcKAS III [36]. Therefore, the binding interactions between ACP and KAS III exhibit species-specific differences. In our further studies, the structure of AbACP and molecular interactions between AbACP and AbKAS III investigated in our current study by NMR spectroscopy will allow us to discover novel potent PPI inhibitors of the *A. baumannii* FAS proteins.

## 3. Discussion

In this study, the structure of *A. baumannii* ACP, which plays a key role in the synthesis of fatty acids, was determined for the first time. The amino acid sequence of AbACP showed high similarity (> 60%) with other Gram-negative bacterial ACPs, as did the negatively charged residues in the “recognition α_2_-helix”, which are important for binding to other FAS enzymes [24,25]. Divalent cations stabilize the ACP structures. Indeed, EcACP and VhACP contain two metal binding sites at either end of the α_2_-helix. Meanwhile, in the absence of divalent metal ions, VhACP does not fold properly due to the absence of a conserved histidine at the α_4_-helix, whereas the A75H mutant undergoes proper folding even in the absence of divalent cations [29]. Additionally, AbACP has a higher melting temperature compared to EcACP in the presence and absence of metal ions, which is likely due to differences in their surface charges. This is because AbACP contains 19 negative and six positive charged residues at a ratio of 3.2, while EcACP has 20 negative and five positive residues at a ratio of 4.0, and VhACP has 20 negative and six positive residues at a ratio of 3.3, with a lack of histidine at the C-terminus. Hence, among these Gram-negative ACPs, AbACP has a relatively low negative-to-positive ratio and subsequent lower electrostatic repulsion, resulting in a higher melting temperature compared to that of EcACP in the absence of metal ions (Figure 3d). Not only stabilizing the structure of ACP but, also, divalent cations play an important role as mediators between ACP and fatty acid synthesis partner enzymes, such as acyl carrier protein phosphodiesterase (AcpH) and acyl carrier protein synthase (AcpS) [12].

In AbACP, the mutation of F47 with Ala resulted in a dramatic decrease in thermal stability, confirming the importance of F47 in hydrophobic packing inside the cavity. F47 at the center of the α_2_-helix in AbACP is not conserved in most ACPs; however, it is observed as F50 in *Thermotoga maritima* ACP (TmACP) [32]. In our previous report, the substitution of Ala for F50 in TmACP was found to cause a decrease in the melting temperature from 100.4 to 90.7 °C. In hyperthermostable TmACP, extremely tight hydrophobic packing occurs, as well as extensive ionic clusters on the surface of the protein [32]; thus, although a single F50A mutation was not capable of causing dramatic changes, it did significantly impact the thermal stability. In fact, in mesophilic AbACP, a single F47A mutation caused a 30-degree reduction in thermal stability, implying that F47 is the key residue for tight hydrophobic packing in AbACP.

The spin–relaxation experiments showed that AbACP has fast time scale motion from picoseconds to nanoseconds, while the four helix regions exhibited more rigidity than the loop regions. The high flexibility of the α_3_-helix and α_3_α_4_ loop regions at the entrance of the cavity in AbACP, with low hNOE values, may allow AbACP to perform its role of sequestering the growing acyl chain. Alternatively, slow motions were observed in F29 and A69, which form the hydrophobic cavity and may be related to the shuttling of the acyl chains. These slow motions require further investigation using acylated ACP with different lengths. 

We also analyzed the interactions between AbACP and AbKAS III to inform the development of the PPI inhibitors. It has been reported that FAS proteins possess a conserved electropositive and hydrophobic surface that interacts with the electronegative and hydrophobic residues in the α_2_-helix of ACP [25,36]. In the case of AbACP, the highly conserved negatively charged surface was important for the recognition of the FAS proteins. In particular, the highly conserved negatively charged residue E42 in the α_2_-_-_helix, as well as D57, E58, and D59 in the α_3_-helix, exhibited large CSPs upon binding to AbKAS III, forming electrostatic interactions in the AbACP–AbKAS III-binding model. Furthermore, hydrophobic residues such as L38 and M45 in the α_2_-helix also had large CSPs, suggesting the formation of hydrophobic interactions with AbKAS III. These results imply that the binding interactions between these two proteins are dominated by both electronegative and hydrophobic residues near the beginning of the α_2_-helix and the negatively charged residues at the α_3_-helix of AbACP. In the AbACP–AbKAS III-binding model, the binding site between AbACP and AbKAS III had opposite surface charges, where the exposed negatively charged residues in the α_3_-helix (D57, E58, and D59) of AbACP interact primarily with the positively charged residues (K258, R257, and K261) near the entrance of the active site of AbKAS III; these interactions, including the α_3_-helix of EcACP, were not observed in the previously reported EcACP-EcKAS III docking model [36]. 

Alternatively, the previously reported binding model calculated for EcACP with *E. coli* KAS III (EcKAS III) by the docking simulation showed electrostatic interactions between the negatively charged residues mainly in the whole α_2_-helix of EcACP (E42, M45, E48, E49, and E50) with EcKAS III. In the EcACP–EcKAS III complex model, the α_2_-helix of EcACP interacts primarily with EcKAS III by electrostatic interactions [36]. In addition, the M45 residue in the α_2_-helix in EcACP binds to EcKAS III (F213) via hydrophobic interactions, which was observed in the AbACP–AbKAS III model as well. However, in the case of the EcACP–EcKAS III model, R7 and E14 in the α_1_-helix of EcACP bind to H222 and K256 of EcKAS III, respectively, whereas, only in the AbACP–AbKAS III model, the negatively charged residues in the α_3_-helix of AbACP bind to AbKAS III. According to our X-ray structure of AbKAS III, we found that F56, conserved only in KAS III proteins with two insertions, participates in π-stacking interactions with the adenosine ring of CoA. In the current docking simulation, we also found that F56 participates in ACP binding by interacting with hydrophobic L38 near the beginning of the α_2_-helix of ACP. Furthermore, K59 near F56, conserved only in KAS III proteins with two insertions, bind ACP at the CoA-binding region via the electrostatic interaction with E42 in the α_2_-helix of AbACP. Further studies are needed to identify the role of the extra inserted sequence in AbKAS III, which may offer important insights in the development of antibiotics targeting AbKAS III specifically. Indeed, differences in the positively charged residues, such as K59, R257, and K258 in the ACP-binding region of AbKAS III compared to those in EcKAS III, may be responsible for the observed differences in the binding models for the ACP–KAS III complex from *E. coli* and *A. baumannii*. Furthermore, since the undesired killing of diverse commensal bacteria by the abuse of antibiotics is a challenging problem, understanding these specific interactions between AbACP and AbKAS III are poised to promote the development of antibiotics with specificity against the MDR *A. baumannii* infection. 

The interaction between FAS and ACP is transient, such that there have been efforts to trap the complex using the covalent modification of FAS by substrate mimetics attached to ACPs [47]. In one of the early reports describing the structure of *E.coli* fatty acid 3-hydroxyacyl-ACP dehydratase (FabA) and the ACP complex, the interactions between the α_2_-helix and α_3_-helix of EcACP with FabA were revealed. The noted conformational changes in the α_3_-helix led to sequestering of the substrate via a switchblade mechanism [23]. Recently, the EcACP–EcKAS I (FabB) complex structure (PDB ID: 5KOF) consisting of a EcKAS I dimer with each monomer crosslinked to a single EcACP, was determined [48]. Similar to the AbACP–AbKAS III complex, the EcACP–EcKAS I complex structure interacts primarily through the top of the α_2_-helix of ACP and KAS I with additional salt bridges from the α_3_-helix. Negatively charged residues such as D36, D3,9 and E48 of the α_2_-helix and hydrophobic residues such as L38, V41, and M45 in the α_2_-helix are involved in the interaction with KAS I. Additionally, negatively charged residue such as D57 in the α_3_-helix participate in the interaction with R45 of EcKAS I [48]. A similar interaction was observed for KAS II (FabF) of *E. coli* [49,50]. In the case of the EcACP-EcKAS II (FabF) X-ray structure (PDB ID: 6OKG), all portions of the α_2_-helix of ACP have interactions with EcKAS II. D36, D39, E42, and E48 of the α_2_-helix have interactions with the positively charged residues of KAS II. Hydrophobic residues such as L38, V41, and M45 of the α_2_-helix also have hydrophobic interactions with KAS II. Furthermore, D57 in the α_3_-helix of EcACP have electrostatic interactions with KAS II similar to the AbACP–AbKAS III complex. However, unlike the EcACP–EcKAS I structure, Q15 in the α_1_-helix and E54 in the α_2_α_3_ loop in EcACP are also involved in the interaction with KAS II [49,50]. Compared to EcKAS I, the top of the α_2_-helix in EcACP provides the primary interaction with EcKAS II. Moreover, the structure of the *E. coli* malonyl-CoA:ACP tranacylase (FabD) and ACP complex revealed the smallest interface among the ACP–FAS complexes, which was augmented by dynamic conformational freedom between the proteins [51]. When we combined these observations, it appeared that the distinct mode of interaction between each FAS and ACP orchestrates the relay of growing fatty acid chains through fatty acid synthesis. Considering that the FAS of *A. baumannii* has not yet been investigated in detail, we plan to elucidate the mode of interactions between FAS enzymes and AbACP in future studies. 

In the current study, as a strategy for combating MDR *A. baumannii*, we chose FAS proteins of *A. baumannii* for developing novel antibiotics, especially AbKAS III and AbACP, which are essential for fatty acid synthesis. For a clear understanding of their structures in solutions, as well as of their binding dynamics, we utilized NMR spectroscopy to determine the solution structure of AbACP and analyzed its interaction with AbKAS III. The unique F47 residues in the hydrophobic cavity play key roles in the thermal stability of AbACP. In addition, the fast time scale motions in the α_3_-helix and α_3_α_4_ loop regions, as well as slow exchanges in F29 and A69, may impact the delivery of the growing acyl chain to various FAS enzymes. Meanwhile, the flexibility observed in AbACP is required for chain flipping, thereby modulating its function as an acyl carrier in bacterial FAS. NMR and docking calculations revealed that the α_2_-helix and α_3_-helix in AbACP form key interactions with AbKAS III, which appear to be species-specific. The results of this study have the potential to facilitate the design of effective PPI inhibitor antibiotics selective against *A. baumannii*.

## 4. Materials and Methods 

### 4.1. Cloning, Expression and Purification of AbACP and AbKAS III

The cDNA of AbACP was cloned into the pET-11a vector (Novagen, Madison, WI, USA) containing an ampicillin resistance and IPTG promoter region. Then, AbACP plasmid were transformed in *E. coli* BL21 (DE3) cells. The expression, purification, and conversion of apo-AbACP to holo-AbACP used the same method in our previous report [31]. AbACP was purified by using HiTrap^TM^ Q FF, Hiload 16/60 superdex 75, and Resource^TM^ Q columns (GE Healthcare, Uppsala, Sweden). The conversion of apo-ACP to malonyl-ACP was produced by the enzymatic reaction from *A. baumannii* ACP synthase and malonyl-CoA (Sigma-Aldrich, St. Louis, MO, USA) in 20-mM Tris-Cl and 5-mM MgCl_2_ buffer (pH8.0) at 25 °C overnight. For NMR experiments, ACP was expressed as ^15^N- and ^13^C-labeled proteins; precultured recombinant cells were inoculated into 500 mL of M9 minimal medium containing 50-mg/L ampicillin and isotope-enriched ^15^NH_4_Cl and ^13^C-glucose (Cambridge Isotope Laboratories, Andover, MA, USA).

To mutate F47 with Ala in AbACP, polymerase chain reaction (PCR) was performed by using the pET-11a vector, which have the acp gene of *A. baumannii*, using the primer pair, 5′-GTTGAACTTGTTATGTCTGCCGAAAATGACTTCGACATCACT-3′ (forward primer) and 5′-AGTGATGTCGAAGTCATTTTCGGCAGACATAACAAGTTCAAC-3′ (reverse primer) at a concentration of 0.2 µM. After 25 cycles of denaturation step at 94 °C for 30 s, annealing at 60 °C for 1 min, and elongation step at 72 °C for 5 min, we transformed the amplified vectors into *E. coli* BL21 (DE3). The expression and purification of the mutant are same as expression and purification of the wild-type protein.

AbKAS III was cloned to the pET-15b vector (Novagen, Madison, WI, USA) to contain the His-tag and thrombin cleaving site. The expression and purification of AbKAS III were described in our previous study [17]. AbKAS III was purified using a 5-mL chelating Sepharose HP column (GE Healthcare, Uppsala, Sweden). Purified AbKAS III was incubated at 25 °C overnight after treating thrombin (Haematologic Technologies, Essex Junction, VT, USA) to remove His-tag. Then, by using a HiTrap^TM^ Q FF column (GE Healthcare, Uppsala, Sweden), AbKAS III was finally purified.

### 4.2. Circular Dichroism (CD) Experiments

The CD experiments were conducted by a J810 spectropolarimeter (Jasco, Tokyo, Japan) with a cuvette in a 1-mm path length. The concentration of AbACP was 50 µM and dissolved in 25-mM 2-(*N*-morpholino)ethanesulfonic acid (MES), 5-mM dithiothreitol, and 5-mM CaCl_2_. The CD spectra were collected from 200 to 250 nm at 0.1-nm intervals. The mean values from 10 scans were processed with J810 software and plotted as the mean residue ellipticity (θ) in deg cm^2^ dmole^−1^. The melting temperature (Tm value) of the proteins were calculated by monitoring the change in the mean residue ellipticities (θ) at a 222-nm wavelength acquired from 15 °C to 95 °C.

### 4.3. NMR Experiments and Assignments 

All NMR experiments are performed with a Bruker Advance 700- and 900-MHz spectrometer (Bruker corporation, Billerica, MA, USA) at the Korea Basic Science Institute (KBSI), Ochang, Korea at 25 °C. Protein samples concentration was 0.5 mM in 25-mM MES, 5-mM CaCl_2_, 5-mM dithiothreitol, 10% D_2_O, and 0.02% NaN_3_ (pH 6.1). For the backbone assignments of AbACP, HNCACB, and CBCA(CO)NH, triple-resonance spectra were used. Additionally, CC(CO)NH, HBHA(CO)NH, and H(CCO)NH spectra were used for the side chain assignments. Residual dipolar coupling (RDC) between two spins in a backbone amide N−H bond were measured by comparing spatially anisotropic dipolar couplings in the solution and gel phase samples. To prepare the gel sample, AbACP molecules were dissolved in a radially compressed polyacrylamide gel, and the partial alignment of the protein was induced, as described previously [31]. In order to collect the distance information between protons in a three-dimensional space, the ^15^N-NOESY (nuclear Overhauser effect spectroscopy) and ^13^C-NOESY experiments were performed using a 150-ms mixing time. All NMR data were processed using NMRPipe [52] and analyzed by the NMRFAM-Sparky [53] program. 

### 4.4. Structure Calculation

The 3D Nuclear Overhauser Effect (NOE) assignment was performed using NMRFAM-sparky [53] to collect the distance constraints between different protons. In addition, a Residual Dipolar Coupling RDC) experiment was carried out for providing angle information about the orientational restraints. The first 20 lowest-energy structures of AbACP were determined by Xplor-NIH-based structure calculations in the PONDEROSA C/S package [33]. Then, all violations containing distance and angle in the 20 lowest-energy structures were analyzed and refined by using PONDEROSA-Analyzer [33]. The final 20 lowest-energy structures of AbACP were evaluated using PSVS [35]. Final coordinates and NOE constraints were deposited in the Protein Data Bank under the accession number 7E42 and Biological Magnetic Resonance Bank (BMRB) ID 36409. Protein structures were drawn using PyMOL (The PyMOL Molecular Graphics System, Version 2.1.0, Schrödinger, LLC., New York, NY, USA). 

### 4.5. Spin-Relaxation Experiments

The NMR spin–relaxation experiments with various forms of AbACP were performed at 25 °C using a Bruker Advance 700-MHz spectrometer and analyzed R_1_, R_2_, and hNOE (heteronuclear NOE) NMR spectra. For the longitudinal (R_1_) and transverse (R_2_) relaxation experiments, relaxation and recycle delays were set to the same values as in our previous report [54]. R_1_ and hNOE spin–relaxation experiments with AbACP were performed at 25 °C using a Bruker Advanced 700-MHz spectrometer. R_2_ experiments were performed at 700 MHz and 900 MHz to confirm to the conformational exchange. For the longitudinal relaxation (R_1_) experiments, relaxation delays were set to 0.01(×2), 0.05, 0.1, 0.2, 0.3(×2), 0.5, 0.8, and 1.2 s. For the transverse relaxation (R_2_) experiments, relaxation delays were set to 0 (×2), 0.01696, 0.03392, 0.05088 (×2), 0.0848, 0.13568, 0.22048, and 0.32224 s. The hNOE experiments were conducted with the recycle delay and proton saturation times of 4.0 and 3.0 s. All relaxation data were processed using NMRPipe and analyzed by NMRFAM-Sparky. 

### 4.6. Measurement of Chemical Shift Perturbations in the ^1^H-^15^N HSQC-TROSY

CSPs and changes in the intensities during titration between malonyl-AbACP and AbKAS III were monitored by ^1^H-^15^N HSQC-TROSY experiments at 25 °C. AbKAS III was titrated to 0.2 mM of ^15^N-labeled malonyl-AbACP in 40-mM potassium phosphate, 10% D_2_O, and 0.02% NaN_3_ (pH 7.0) with molar ratios of 0, 0.1, 0.5, 1.0, and 1.5, respectively.

### 4.7. Molecular Docking Simulation

The binding model of AbACP and AbKAS III was constructed by AutoDock using the YASARA software program version 20.7.4 (YASARA Bioscience, Wien, Austria) [55]. The crystal structure of AbKAS III (5YO9) was used as a receptor, while the solution NMR structure of AbACP (7E42), determined in this study, was used as a ligand. For the docking simulation, we attached a partial prosthetic group ((3-hydroxy-2,2-dimethyl-4-oxo-4-[methylamino]butyl) phosphate) to S37 on AbACP to penetrate into the active site of AbKAS III. During the docking simulation, all atoms were fixed in AbACP, except for the side chains of the residues such as S37, L38, E42, M45, D57, and E58, which showed CSPs larger than 0.07 ppm upon titration of AbKAS III to malonyl-AbACP. In the case of AbKAS III, we also released the side chains of R257, F260, and M297, as well as the conserved positively charged residues such as K59, K258, and K261, flexibly near the active site in AbKAS III to enable the 4-PP prosthetic group to penetrate into the active site. After 100 docking runs, the resulting model was subjected to 200 steps of the steepest descent minimization method using GROMACS (Groningen Machine for Chemical Simulation; University of Groningen, Groningen, The Netherlands). We selected the best docking model with the lowest binding energy and highest population, where the 4-PP prosthetic group penetrated into the active site of AbKAS III.

## 5. Conclusions

In conclusion, we determined the solution structure of AbACP and analyzed the structural and dynamic properties of AbACP. The unique F47 residue forming hydrophobic packing in the hydrophobic cavity plays a key role in the thermal stability of AbACP. NMR and docking calculation revealed that α_2_-helix and α_3_-helix in AbACP form key interactions with AbKAS III. Since these interactions are different between the species, the information provided in this study can be the initiative to design effective PPI inhibitors, such as effective and selective antibiotics against *A. baumannii*. In addition, we found that the fast time scale motions in the α_3_-helix and α_3_α_4_ loop regions, as well as slow exchanges in F29 and A69, may play important roles for the delivery of growing acyl chains to various FAS enzymes. The flexibility observed in AbACP is required for chain flipping to play an important role in modulating its function as an acyl carrier in bacterial FAS. Our findings are important groundwork for the further design of PPI inhibitors to target *A. baumannii* FAS proteins, since MDR *A. baumannii* is an emergent problem.

## Figures and Tables

**Table 1:** Statistics of the 20 lowest energy structure of *Acinetobacter baumannii* acyl carrier protein (AbACP) (Protein Data Bank(PDB) ID: 7E42).

Restraints ^a^	
Total	1465
Conformationally restricting distance constraints	
Short Range [(i − j) ≤ 1]	157
Medium Range [1 < (i − j) ≤ 5]1	573
Long Range [(i − j) ≥ 5]	446
Dihedral angle constraints	
Phi	71
Psi	70
Residual dipolar coupling constraints	74
Hydrogen bond constraints	74
**Average RMSD (** **Root Mean Square Deviation)** **to the Mean Xplor-NIH Coordinates (Å) ^b^**	
Backbone atoms (all residues/order residues) ^c^ Heavy atoms (all residues/order residues) ^c^	0.1/0.1
	0.4/0.4
**Summary of Ramachandran Plot from PROCHECK (%) ^b^**	
Most favored regions	97.6
Additionally allowed regions	2.4
Generously allowed regions	0
Dis-allowed regions	0
**Average Number of Violations per Xplor-NIH Conformer ^d^**	
Distance constraint violations (>0.2 Å)	0
Angle constraint violations (>10°)	0

^a^ The structure of holo-AbACP was calculated by using Xplor-NIH-based calculations in PONDEROSA C/S [33]. ^b^ The final 20 lowest energy structures were evaluated by Protein Structure Validation Software suite (PSVS) [34]. ^c^ Ordered residues are from D3 to G78. ^d^ All violations of residues are analyzed by using PONDEROSA-Analyzer [35].

## Supplementary Materials

The following supplementary materials can be found at https://www.mdpi.com/1422-0067/22/7/3317/s1.
