# Supplementary material for: Deciphering the Binding Interactions between Acinetobacter baumannii ACP and β-ketoacyl ACP Synthase III to Improve Antibiotic Targeting Using NMR Spectroscopy
Source: Int J Mol Sci. 2021 Mar 24;22(7):3317. doi: 10.3390/ijms22073317 (PMC8036411; doi:10.3390/ijms22073317)
Supplement: Supplementary file 1 [file ijms-22-03317-s001.pdf]

International Journal of *Molecular Sciences*

MDPI

Supplementary Information for an Article Titled

Deciphering the Binding Interactions between *Acinetobacter baumannii* ACP and β-ketoacyl ACP Synthase III to Improve Antibiotic Targeting

Sungjae Choi, Jungwoo Park, Jiwon Yeon, Ahjin Jang, Woo Cheol Lee, and Yangmee Kim *

Department of Bioscience and Biotechnology, Konkuk University, Seoul 05029, South Korea; csj9897@konkuk.ac.kr (S.C.); jhopark123@konkuk.ac.kr (J.P.); jiwon4533@konkuk.ac.kr (J.Y.); ajin931017@konkuk.ac.kr (A.J) wclee3@konkuk.ac.kr (W.L.)

* Correspondence: ymkim@konkuk.ac.kr; Tel: +822-450-3421; Fax: +822-447-5987

Citation: Choi, S.; Park, J.; Yeon, J.; Jang, A.; Lee, W.C.; Kim, Y. Deciphering the binding interactions between *Acinetobacter baumannii* ACP and β-ketoacyl ACP synthase III to improve antibiotic targeting using NMR spectroscopy. *Int. J. Mol. Sci.* **2021**, *22*, 3317. https://doi.org/10.3390/ijms22073317

Academic Editor: Congbao Kang

Received: 22 February 2021
Accepted: 21 March 2021
Published: 24 March 2021

Publisher's Note: MDPI stays neutral with regard to jurisdictional claims in published maps and institutional affiliations.

Copyright: © 2021 by the authors. Licensee MDPI, Basel, Switzerland. This article is an open access article distributed under the terms and conditions of the Creative Commons Attribution (CC BY) license (http://creativecommons.org/licenses/by/4.0/).

Figure S1. The chemical shift perturbations of AbACP in 1H-15N HSQC spectra upon 4'-phosphopantetheine group attachment at S37 of apo-AbACP to form holo-AbACP.

Table S1. Binding interactions between AbACP and AbKAS III in the lowest energy docking model.

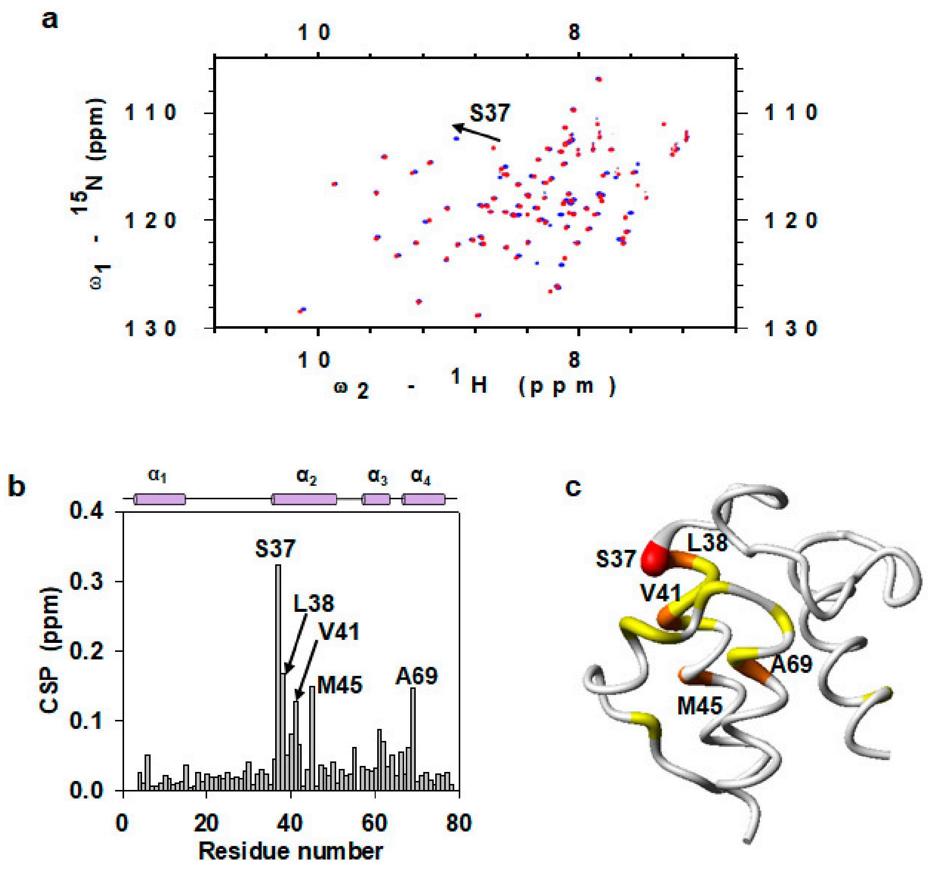

Figure S1. The chemical shift perturbations of AbACP in ^{1}H-^{15}N HSQC spectra upon 4′-phosphopantetheine group attachment at S37 of apo-AbACP to form holo-AbACP. (a) Overlay of the ^{1}H-^{15}N HSQC spectra for apo-AbACP (red) and holo-AbACP (blue). (b) Chemical shift perturbations induced by 4′-phosphopantetheine group modification. Chemical shift perturbations (Δδ) are calculated using the equation: $\Delta\delta=(0.5(\Delta\delta(^1H)^2 + (\alpha\Delta\delta(^{15}N)^2)))^{1/2}$ (α = 0.2 for most residues and α = 0.14 for glycine). (c) Residues showing large chemical shift perturbations with worm notation. (Red, CSP > 0.2 ppm; orange, CSP > 0.1 ppm; yellow, CSP > 0.05 ppm)

Table S1. Binding interactions between AbACP and AbKAS III in the lowest energy docking model

	Binding interactions in docking model between AbACP and AbKAS III			Residues with large CSPs of amide protons> 0.07ppm corresponding to the binding interactions and their CSPs in ppm
	AbACP	AbKAS III	Distance (Å)	
H-bond	E42(OE2)	K59(HZ1)	2.6	E42 (0.071)
	E42(OE1)	N296(HD22)	2.3	E42 (0.071)
	D57(OD1)	R257(HH12)	3.2	D57 (0.11)
	D57(OD2)	K258(HZ3)	2.2	D57 (0.11)
	E58(OE2)	K261(HZ1)	2.4	E58 (0.14)
	E58(OE2)	K261(HZ2)	3.0	E58 (0.14)
	S37(PP-N41)	Q254(HE12)	2.6	S37 (0.11)
	S37(PP-H36)	N294(ND2)	2.5	S37 (0.11)
Electrostatic interaction	E42(OE2)	K59(HZ1)	2.6	E42 (0.071)
	D57(OD1)	R257(HH12)	3.2	D57 (0.11)
	D57(OD2)	K258(HZ3)	2.2	D57 (0.11)
	E58(OE2)	K261(HZ1)	2.4	E58 (0.14)
Hydrophobic interaction	L38(CD1)	A60(CB)	3.6	L38 (0.21)
	M45(CE)	F260(CE2)	3.5	M45 (disappeared)
	S37(PP-C31)	R257(CD)	3.5	S37 (0.11)
